# Illness perceptions in the context of differing work participation outcomes: exploring the influence of significant others in persistent back pain

**DOI:** 10.1186/1471-2474-14-48

**Published:** 2013-01-30

**Authors:** Joanna Brooks, Serena McCluskey, Nigel King, Kim Burton

**Affiliations:** 1 Centre for Applied Psychological Research, Institute for Research in Citizenship and Applied Human Sciences, University of Huddersfield, Queensgate, Huddersfield, HD1 3DH, UK; 2 Centre for Health and Social Care Research, Institute for Research in Citizenship and Applied Human Sciences, University of Huddersfield, Queensgate, Huddersfield, HD1 3DH, UK

## Abstract

**Background:**

Previous research has demonstrated that the significant others of individuals with persistent back pain may have important influences on work participation outcomes. The aim of this study was to extend previous research by including individuals who have remained in work despite persistent back pain in addition to those who had become incapacitated for work, along with their significant others. The purpose of this research was to explore whether the illness beliefs of significant others differed depending on their relative’s working status, and to make some preliminary identification of how significant others may facilitate or hinder work participation for those with persistent back pain.

**Methods:**

Interviews structured around the Illness Perception Questionnaire (chronic pain version) were conducted with back pain patients recruited from a hospital pain management clinic along with their significant others. Some patients had remained in work despite their back pain; others had ceased employment. Data were analysed using template analysis.

**Results:**

There were clear differences between beliefs about, and reported responses to, back pain symptoms amongst the significant others of individuals who had remained in employment compared with the significant others of those who had ceased work. Three overarching themes emerged: perceived consequences of back pain, specific nature of employment and the impact of back pain on patient identity.

**Conclusions:**

Significant others of employed individuals with back pain focused on the extent to which activity could still be undertaken despite back pain symptoms. Individuals out of work due to persistent back pain apparently self-limited their activity and were supported in their beliefs and behaviours by their significant others. To justify incapacity due to back pain, this group had seemingly become entrenched in a position whereby it was crucial that the individual with back pain was perceived as completely disabled. We suggest that significant others are clearly important, and potentially detrimental, sources of support to individuals with back pain. The inclusion of significant others in vocational rehabilitation programmes could potentially be a valuable way of mobilising readily accessible resources in a way that supports optimal functioning.

## Background

Back pain is often a self- limiting condition, with most cases resolving within six weeks of symptom onset [1]. However, for the substantial minority of people (around 10%) who have not recovered after twelve weeks, and who develop persistent pain, the long-term prognosis is often uncertain, and personal, financial and societal costs associated with the condition can be substantial [2]. Since remaining in work (or returning to work as soon as possible) limits the potentially negative social, psychological and physical effects of long term sickness absence [3,4], identifying obstacles to work participation is an important goal [5].

It is widely accepted that psychosocial factors are important determinants for the consequences of back pain, including the transition to chronicity, (e.g. [6-9]). However, it remains unclear which specific factors are of particular relevance in this context, and a comprehensive understanding of how pertinent psychosocial factors might operate in determining functional outcomes (including work participation) is still required. There have been several recent calls in the literature for more qualitative work in this area as the most suitable method through which to explore psychosocial risk factors for chronicity [10] and occupational outcomes [11].

It is becoming apparent that what individuals believe about their symptoms and the meanings they attach to these (their ‘illness perceptions’ ‘illness beliefs’ or ‘illness representations’) may be particularly important for both clinical and occupational outcomes in low back pain (e.g. [8,12-16]). The Common Sense model of self-regulation (CSM) (e.g. [17]) is a theoretical model established as a useful framework through which to elicit and explore illness perceptions. The CSM conceptualises individuals as having internal common sense models about perceived health threats, which in turn impact on the coping strategies adopted by individuals to manage their symptoms. To date, there has been little in-depth research on the influence of illness perceptions on persistent back pain and work participation. Furthermore, research in this area tends to focus on individual illness perceptions, yet it has been suggested that the significant others (spouse/partner/close family member) of individuals with persistent pain may have an important mediating influence on illness course and occupational outcomes [18-22]. Previous research suggests that significant others are salient sources of discriminative cues, punishment or reinforcement for pain behaviours [18-20], and that spousal pain beliefs about disability, treatment control and medication are significantly correlated with partners’ pain severity and other indicators of pain adjustment [23]. Significant others’ behavioural responses have additionally been shown to be associated with patient outcomes in other chronic illness conditions [24].

A recent qualitative study in the United Kingdom exploring the illness perceptions of those claiming state disability benefit due to persistent back pain and their significant others highlighted the importance of significant others and an individual’s wider social circumstances on recovery and work participation [25]. To date, there has been little consideration given to how significant others might usefully be involved in interventions to improve work participation for those individuals with persistent back pain, and therefore the present study sought to further extend this area of research by also including individuals who have remained in work despite persistent back pain in addition to those who had become incapacitated for work, along with their significant others. This allowed a more in-depth exploration of whether the illness beliefs of significant others differed depending on their relative’s working status, and to make some preliminary identification of how significant others may facilitate or hinder work participation for those with persistent back pain.

## Method

### Study design

Individual semi-structured interviews were conducted using an interview schedule designed to elicit illness perceptions along the dimensions delineated in the CSM. This theoretical framework incorporates both cognitive and emotional representations of illness along five core dimensions: illness identity, perceived causality, expectations about timeline, consequences of illness, and beliefs about curability and control. An existing, well-validated quantitative measure of illness perceptions in chronic pain conditions (the revised Illness Perception Questionnaire, chronic pain version, [26]) was used to develop open-ended questions to encourage participants to reflect on and fully elucidate their experiences and beliefs in relation to the back pain condition and work. This research design has been successfully employed in previous work and further information on the study design can be found elsewhere [25,27]. Relevant permissions for the study were obtained from National Health Service Research Ethics (reference number 11/H1302/6).

### Participants

A convenience sample of patients reporting non-specific low back pain of at least twelve weeks duration were recruited from a hospital pain management clinic in northern England. The employment status of patients was recorded at point of entry to the study, and patients who were not currently in employment had to attribute their lack of work participation to their back problem to be eligible for participation. Patients meeting the study criteria, and who had a significant other, were identified and recruited by the hospital consultant running the pain clinic. Participants were given full study information sheets and written informed consent obtained.

The mean age of working patients was 49.2 years (ranging from 45 to 52 years) and the mean age of their significant others was 36.6 years (ranging from 25 to 48 years). Amongst patients who were not working, the mean age was 57 years (ranging from 51 to 63 years), and the mean age of their significant others was 61.5 years (ranging from 57 to 68 years). All participant dyads in the non-working sample were spousal relationships; amongst the working dyads, three were in spousal relationships and two were parent/ adult child dyads (in both cases the significant other was the adult child). All participants identified their ethnic background as ‘White British’. Details of our participant sample, including the present employment status and past employment details for both patients and significant others, are presented in Table 1.

**Table 1:** Participant details

***Name of patient (Pseudonym)***	***Name of significant other (Pseudonym)***	***Relationship to patient***	***Patient employment status***	***Years in education (patient)***	***Years in education (significant other)***	***Significant other occupation***	***Duration of patient back pain***
Sarah	Harry	Spouse	Out of work (previously supermarket checkout assistant)	Left at 16	Left at 16	Engineer	11 years
Mick	Belinda	Spouse	Out of work (previously bus driver)	Left at 16	Left at 16	Unemployed (stopped work to care for patient)	18 months
Elizabeth	Frank	Spouse	Out of work (previously school cleaner)	Left at 16	Left at 16	Retired	3 years
Hannah	Gary	Spouse	Out of work (previously clerical worker)	Left at 16	Left at 16	Council worker (manual)	5 years
Sam	Tess	Spouse	Employed (Manager)	Degree	Doctoral	Doctor	11 years
Rob	Vikki	Spouse	Employed (Manager)	Degree	Professional qualification	Management consultant	10 years
Sally	Will	Son	Employed (volunteer service)	Left at 16	Left at 16	Property developer	26 years
Elena	David	Spouse	Employed (Training consultant)	Degree	Degree and professional qualification	Teacher	3 years
Ruth	Brian	Son	Employed (Social worker)	Professional qualification	NVQ	Plumber	3 years

A total of eighteen interviews were undertaken by JB, SM and NK. Interviews lasted around one hour and participant dyads were interviewed separately at a time and place convenient to them. Interviews were digitally recorded and transcribed verbatim. The following areas were covered in each interview: (1) history of the illness (‘perceived causality’ dimension of the CSM); (2) perceptions of identity of the illness and current status of the illness, including symptoms (‘illness identity’ dimension); (3) illness management (‘beliefs about curability and control’ dimension); (4) timeline of the illness (‘expectations about timeline’ dimension); (5) impact of the illness on the lives of both patient and spouse (‘consequences of the illness’ dimension). The interview schedule was flexible and allowed participants to raise topics as they wished, assisting in the establishment and maintenance of rapport. Questions were open-ended and non-directive, and were modified to be posed to the patient or their spouse as appropriate. Full copies of the interview schedule are available from the corresponding author on request.

### Data analysis

Interviews were analysed using the template analysis style of thematic analysis [28], a systematic technique for categorising qualitative data in hierarchical clusters. This technique has been previously used in both healthcare and occupational research [28]. Template analysis allows for the definition of ‘a priori’ themes (aspects of the phenomena under investigation that are of particular interest) in advance of the analysis process [29]. Those a priori themes that do not prove useful in representing and capturing key messages are redefined or discarded as the template is modified in the process of data analysis. New themes, emerging through analytic engagement with the data, may be defined and added to the template structure. In the present study, the use of this technique allowed for analysis to be guided by and initially structured around our research focus on the dimensions of the CSM, a model which is already well- established as a useful framework for exploring beliefs about illness and therefore a logical starting point for this exploratory work. Through the process of analysis we were additionally able to identify and incorporate into our template structure additional themes representing some of the ways in which other factors and beliefs may impact on work outcomes for patients with chronic back pain.

The main procedural steps undertaken in our analysis were as follows: (1) We thoroughly familiarised ourselves with our interview data through reading and re-reading of the interview transcripts; (2) we carried out preliminary thematic coding of the data using the CSM framework to tentatively define a priori themes, whilst additionally recording any new themes which emerged from the data and which appeared interesting and relevant; (3) we organised our emerging themes into meaningful clusters and began to define an initial coding template incorporating the relationships between and within themes; (4) we applied our initial template to our interview data and modified the template in an iterative process until all members of the research team were satisfied that the template provided a comprehensive representation of our interpretation of our interview data. Our initial analysis drew on the template designed and applied in our earlier work [25] and we found that it successfully incorporated the data derived from the current study, although it required structural modification at lower thematic levels, with some themes being retitled and amended to better represent the key messages derived from interview data (see [27] for further detail). All interview data were mapped onto the template and the iterative process of data analysis continued until all relevant data had been satisfactorily coded to the modified template. Initial analysis was undertaken by JB and SM, NK and KB checked the coding and analysis and the final template was agreed by all authors.

Our final coding template was comprised of both themes derived from the CSM and themes which emerged through analysis of our interview data and is available from the corresponding author on request. For the purposes of the present article, we focus on three themes which incorporate data suggesting clear distinctions between dyads in which patients had remained in employment and dyads in which the patient was no longer in work due to their back problem – (1) Consequences of illness; (2) Nature of work and (3) Patient identity. The first of these (consequences of illness) is a theme delineated in the CSM, the latter two themes emerged through our engagement with our participants’ interview data. For example, according to the CSM, ‘illness identity’ pertains to the specific symptoms associated by a patient with an illness. Our ‘patient identity’ theme highlights ways in which beliefs about personal characteristics may additionally play an important role in response to and management of symptoms. Our analysis demonstrates how in-depth qualitative research can usefully extend and explicate existing theory, allowing better understanding of respondents’ perspectives and beliefs, and allowing additional dimensions not incorporated in standard quantitative assessment of illness representations to emerge from the data. Verbatim extracts from interviews are presented to illustrate our findings, and pseudonyms are used throughout.

## Results

The three themes on which we will focus for the purposes of this article are themes in which there were apparent differences between our working and non-working samples. An overview of the themes along with specific examples is presented in Table 2, and each theme is then presented, defined and discussed at greater length.

**Table 2:** Definition of themes and examples

**Theme title and definition**	**Example quotation from working sample**	**Example quotation from non-working sample**
**1. Consequences of illness**	*“It doesn’t impact on anything, ‘cos he doesn’t not do anything because he’s got pain”*	*“Like making a cup of tea, I’ll say ‘Are you alright, be careful’, I say, ‘Can I help?’ and I know I can’t help at all”*
Extent to which participants reported that the patient’s back pain impacted on their everyday life and activities		
**2. Nature of work**	*“My immediate line manager is very supportive”*	*“There weren’t really a lot they could find she could do”*
Ways in which the nature of patient participants’ particular professions were reported as impacting on whether or not patients had been able to continue in employment with back pain.		
**3. Patient identity**	*“He has this determination where he won’t let it, he won’t give in to it and he won’t let it beat him”*	*“His workmates thought ‘There’s nowt wrong with you, you’re sat down, you’re walking around’, but once he’s sat down, he’s got pain but they can’t see that”*
Refers to participants’ depictions of the patient in relation to their self-management of their back pain condition – hero or victim narrative?		

### Consequences of illness

This theme refers to the extent to which participants reported that the patient’s back pain impacted on their everyday life and activities. In the employed sample, when asked about the consequences of the patient’s back pain, both significant others and patients focused on how the patient did not allow their back pain to prevent them from undertaking activities. They emphasised what the patient could do despite their back problem, rather than what they were unable to do.

“In terms of what does it impact on, well it doesn’t impact on anything, ‘cos he doesn’t not do anything because he’s got pain. He’s definitely not sitting around not doing anything going ‘I’ve got a back problem’. He gets fed up with it, but it’s not really stopped him”

[Tess – significant other, working sample]

“I live with chronic pain. But me and my pain are like hand in glove. And I’m the glove. I’m always on top of the pain. When I knew that it wouldn’t get any better, that I had to live with this pain at this level or different levels, as it may be depending, I decided to resume work”

[Sally – patient, working sample]

In contrast, patients and significant others from the out of work sample emphasised the far-reaching consequences of the back pain on the minutiae of everyday activities.

“Going to supermarket, we’ve got to go together now. Before she’d go on her own, but now we’ve to go together. Because she’s got trolley to hold onto she’s alright, but a loaf of bread and one or two other bits in the bag and that’s it. But four pints of milk, you know, she can’t pick more than one up, so we’ve to go together”

[Harry – significant other, non-working sample]

“I always feel conscious that I’m overloading other people, you know, just like making a cup of tea, I’ll say ‘Are you alright, be careful’, I say, ‘Can I help?’ and I know I can’t help at all, I can’t.”

[Sarah – patient, non-working sample]

Significant others in the non-working sample were described as taking on a role in which they acted as anxious bystanders, with both patients and significant others in this group tending to ‘catastrophise’ regarding potential rather than actual consequences of the condition.

“I can’t leave him to walk up town on his own. Crossing roads he’s really slow, all it takes is one car through a red light, you know. It does frighten me so I don’t leave him on his own”

[Belinda – significant other, non-working sample]

“The wife says to me, ‘You’re smelling a bit, why aren’t you showering? Just cos you’re ill doesn’t mean you can’t shower’. I says, ‘The trouble is love’, I says, ‘The problem is, I’m frightened of getting in the shower’. She says ‘Why?’ I says ‘I’m frightened, I don’t want to fall’. She says, ‘You silly bugger, why didn’t you tell me?’ So when I shower now, she’s stood there with me at the side of me so she can get hold of me if anything happens”

[Mick – patient, non-working sample]

However, close reading of the interview data revealed that participants did not report patients in the non-working sample as actually physically unable to undertake activities due to their condition. In fact, there were very few examples of activities that patients were reported as completely physically unable to undertake, and those that were highlighted, whilst impacting on quality of life, did not constitute activities that could be defined as ‘essential’:

“She has to sit out once or twice at line dances”

[Frank – significant other, non-working sample]

“When we were on holiday, I couldn’t even get comfortable on the sunbed, I couldn’t even lie on my back for ten minutes”

[Hannah – patient, non-working sample]

Bluntly put, patients who were out of work were, at least to some extent, apparently self-limiting, fearful that activity of any kind would exacerbate their condition and were supported in these beliefs by their significant others. However, deeper analysis of the interview data at more detailed lower level coding allows for further exploration of the development of and reasoning behind these ostensibly unhelpful beliefs.

### Nature of work

This theme refers to ways in which the nature of patient participants’ particular professions were reported as impacting on whether or not patients had been able to continue in employment with back pain. Participants in the working dyads acknowledged that patients had not carried on in employment entirely unaffected by their back problem. Flexibility from employers, primarily in allowing time off to attend medical appointments but also allowing for reduced or flexible working hours in some instances, was vital in facilitating continued employment. Regrettably, even amongst those patients who had managed to continue in work, this was not always described as easily forthcoming.

“Earlier this year actually the HR department were starting to ask questions around his time off for his back injections … I cover HR as part of what I do, and I basically said ‘Get back to work and tell them to get lost and we’ll take a claim against them if they try this one because the only reason you can go to work is because your pain is managed for you and you’ve worked for them for bloody donkey’s years’…The trouble is it’s not necessarily the people you’re working with, it’s someone over there who doesn’t know you from Adam, who’s making decisions about you who doesn’t know the full picture”

[Vikki – significant other, working sample]

Both patients and their significant others had usually been involved in the process of negotiating and maintaining necessary concessions at work; often it was good personal relationships with line managers that facilitated these arrangements.

“At that time he had a line manager who was very sympathetic. I don’t think anybody’s been allowed to do it since or before then. But I wrote his application form, maybe that helped”

[Tess – significant other, working sample]

“My immediate line manager is very supportive and the guy who was my boss at the time that I started with this was extremely supportive. It tends to be the ones above, you know the next line up, they’re not that supportive, it’s like ‘Yeah, whatever’ but yeah, my immediate line management have been fantastic”

[Sam – patient, working sample]

It was evident that participants in this sample were well-informed about their rights and the employer’s responsibilities and were confident of the patient’s value and worth to the employer.

“Even if I went off sick, if I had to have time off to have this spinal operation, I would expect after the amount of years I’ve worked for them, I would blooming well expect them to pay for me to be off and to hold my post”

[Elena – patient, working sample]

In contrast, participants in the non-working sample described the nature of the patient’s previous employment as wholly incompatible with the patient’s back problem.

“She can’t sit for too long before she’s to get up to move around and she can’t stand for long before she needs to sit down. What job is there that line of work? She can’t sit at the check-out with twisting, at the cigarette kiosk you’re stood up and moving around, so there weren’t really a lot they could find she could do”

[Harry – significant other, non-working sample]

“Initially I carried on at work and they sort of got somebody to lift chairs up for me, you know, so that I wasn’t lifting heavy things and things like that. They did at the beginning, but they’re not going to do it all the time, they wouldn’t, and I wasn’t going to ask them, you know. I’d have felt inadequate, I’d have felt that they were saying ‘She’s come back and she can’t even do this and can’t even do that’, so I didn’t even go down there”

[Elizabeth – patient, non-working sample]

Participants did not necessarily perceive the patient’s previous employer as unsympathetic or in any way at fault, but neither patients nor significant others perceived the patient as having any rights or recourse to action in this context, in direct contrast to the working sample, who were articulate, confident and informed around the patient’s employment rights.

For patients who were not working and their significant others, circumstances over which patients were perceived to have little control were thus reported to have contributed to their inability to both continue in employment and benefit from the positive psychological advantages afforded by continued employment. Participants in the working sample did not attribute the patient’s continued employment to economic necessity but described the beneficial consequences of employment in terms of positive self-identity and as a welcome distraction from the back problem.

“I think the work’s good for her because it gets her out and about”

[Will – significant other, working sample]

“He goes to work because he just won’t give in to it and because he wants to keep him himself occupied. He says ‘I’m not an invalid and I’m not going to give in to it’”

[Vikki – significant other, working sample]

“I’ve found the best treatment for the back pain is me getting on with work, getting on with life”

[Rob – patient, working sample]

Both patients and significant others in the non-working sample were resigned to the permanent effects of the patient’s back problem on their employment status and were thus more likely to consider the patient as ‘disabled’, a role which might become self-fulfilling. Our data suggest that the ability of participants to remain in employment was in part influenced by the nature of their work (whether or not adaptations could be made to enable employees to continue in post despite their symptoms) and in part due to patients’ confidence and ability to negotiate adaptations with their employers (significant others often described themselves as being an important source of support for the patient in this context). Whilst the patients in our ‘out of work’ sample had not necessarily worked in manual jobs, the nature of their previous roles could be perceived as having limited scope for adaptations to accommodate their back problem. In our ‘working’ sample, patient participants had higher status roles in which the work involved was described as more easily allowing for some balance between sedentary positions and physical movement. These participants had been able to negotiate flexible working hours and adaptations to their role where they felt this necessary.

### Patient identity

This theme refers to participants’ reflection on personal character traits of the patient in relation to their self-management of their back pain condition. Although our analysis identified socio-economic factors including nature of work as an area in which there seemed to be clear differences between the two samples, potentially accounting to some extent for differing work participation outcomes, interestingly participants themselves did not identify these as accounting for patients’ employment status. Both patients and significant others were more concerned to explain the patient’s current functional status in terms of personal narratives relating to the patient as either stoical and heroic in the case of the working sample or as a blameless victim amongst the non-working sample. This focus on individual character traits seemed to arise at least in part in response to perceptions of outsiders’ attitudes towards the patient and their condition. It is known that patients with back pain often perceive themselves as stigmatised due to their condition [30] and it has been suggested that significant others may attempt to support patients through verbal narratives providing witness to and validation of patient incapacity in the face of perceived outsider scepticism [25]. Our interview data showed that, across the two sample types, significant others reported that their closeness to their relative afforded them the opportunity to witness the true impact of the back pain in a way that outsiders could not due to the invisibility of the pain symptoms.

“His workmates thought ‘There’s nowt wrong with you, you’re sat down, you’re walking around’, but once he’s sat down, he’s got pain but they can’t see that, they didn’t see that”

[Belinda – significant other, non-working sample]

“She covers it up so people don’t realise, everyone thinks she’s alright, but she’s not, she’s putting on a brave face, I know”

[Brian – significant other, working sample]

However, the two samples notably differed in their portrayal of the patient. Amongst the working sample, both patients and significant others firmly rejected any notion of the patient being disabled by their condition. Rather than seeing the patient as a victim, participants in this group used the back pain as evidence of the patient’s strength of character.

“I think she herself manages remarkably. I think she does what she can and I think she’s managed it really well”

[David – significant other, working sample]

“He’s incredible really. He has an amazing pain threshold. He can push that pain threshold up to another level and he must do that psychologically because why is he different to anybody else? It can only be a mind over matter thing can’t it and how he has this determination where he won’t let it, he won’t give in to it and he won’t let it beat him and he doesn’t want to give in”

[Vikki – significant other, working sample]

With no interviewer prompting, every significant other participant from the working sample drew some comparison between their family member who had maintained their employment despite their back pain and others with back pain who had ceased employment. All such comments tended to be somewhat disparaging regarding those who reported themselves as unable to work due to back pain.

“I think it’s frustrating, loads of people just ‘Yes, but’, all going ‘I can’t do that’, ‘I haven’t tried that’, ‘I’m not doing that’ … Frustrating because there’s people as well, people who have a really chronic difficult illness trying hard and people who don’t appear to be that incapacitated going ‘I can’t possibly do this’

[Tess – significant other, working sample]

Participants in the non-working sample were well aware of the potentially censorious attributions associated with being out of work due to back pain especially in relation to claiming state benefits, and significant others in this sample were particularly vociferous in their defence of the patient, railing against others’ lack of understanding of the patient’s condition.

“We’ve never claimed incapacity and we’ve never claimed any injury, which ninety percent of people would, you know, and that isn’t an issue, you know, she’s got it and that’s it, and now she just, she wants to get the best out of life”

[Gary – significant other, non-working sample]

“He was wrongly accused of something, somebody wrote to them to say he was claiming disability fraudulently and they were investigating for fraud, they sent a letter to his supervisor asking if he knew anything about his disability . It makes me so angry, they took his car off him, the disability living allowance off him, and now we’re having to fight for them back again”

[Belinda – significant other, non-working sample]

From the above analysis it is understandable how patients and particularly significant others in the non-working sample would resort to a ‘patient as truly disabled’ narrative. These participants view patients as unfairly stigmatised as potential malingerers, and perceive themselves as lacking in personal control over their – or their significant other’s - employment situation. It is therefore not surprising that they place a strong emphasis on the serious and far-reaching consequences of the patient’s back problem.

## Discussion

Significant others are thought to have an influential role in the experience and progression of back pain, yet little research has thus far examined this role in the context of work participation. Findings from this exploratory study provide some potentially useful insights into the ways in which both individuals with persistent back pain and their significant others conceptualise and respond to back pain. The inclusion of significant others highlights some interesting and currently under-researched areas relating to wider social circumstances, which may play an important role in influencing occupational outcomes. It follows that these factors are potentially important in the design and implementation of occupational rehabilitation programmes.

There was a notable difference in the way that participants from the working and non-working samples described how the patient’s back condition had impacted on both the patient’s identity and on their activities. Significant others from the working sample tended to emphasise what the patient could do despite their condition and attributed this to the patient’s admirable personal characteristics, describing them as heroic and stoical. In contrast, our non-working sample emphasised the extent to which the condition prevented the patient doing things and descriptions of patient identity focused on the patient as a helpless victim, anticipating and rebuffing potential accusations of personal responsibility and blame. For significant others of patients out of work due a back problem, it may be that to justify the patient’s inability to continue in employment, it is necessary that they be defined in terms of their incapacity and in terms of their being ‘disabled’. This means emphasising what patients cannot do rather than what they can do, with potentially detrimental effects on their activity and identity. In the face of stigmatising socio-cultural beliefs about ‘benefit cheats’ and ‘malingering’ [31], significant others may feel they cannot allow room for scepticism to develop and it is therefore important that they support their ‘other’ by emphasising their inactivity and/or disability. This is likely to have negative consequences in terms of participation outcomes because activity avoidance is in direct opposition to the clinical guidelines for best practice management of persisting low back pain, which recommend that people with low back pain should be advised that staying physically active is likely to be beneficial [32].

Whilst work is generally good for health and wellbeing, there is a recognised social gradient in health partly dependent on the type of work [3]. For participants in our ‘non-working’ sample, the nature of their previous roles could have limited scope for workplace adaptation, and it has been documented that particular difficulties exist in any return to work process after a period of ill health for individuals with lower levels of educational achievement and often physically demanding jobs [25,33]. By contrast, our ‘working’ participants had higher status roles in which the work involved was described as more flexible, and participants had been able to negotiate their working hours and adaptations where they felt this necessary. Further examination of the data revealed that the working participants often felt it was a personal relationship with line management that had made these concessions possible, with Human Resource departments often described as less helpful and impersonal. These findings provide additional support for the notion that line managers have a key role in this context, which warrants further exploration to ensure that those undertaking this role have the necessary knowledge and support [34,35]. Further research might also usefully focus on identifying how the design of interventions might assist in helping individuals stay economically active when the nature of a job role or business presents less obvious opportunities for redeployment or retraining. Whilst certain work may be perceived by both employer and employee as being limited in scope for adaptation, with some inventiveness in identifying and overcoming the pertinent obstacles, accommodations may in fact be possible [35]. More recent work emerging from the body of literature on organisation culture and reflecting on how workplaces can best foster a supportive culture to overcome workplace obstacles may be useful in this respect [3]. Others have highlighted the importance of making sure that employees are fully informed about their rights and responsibilities in the context of musculoskeletal pain conditions [36] and the present study lends further weight to this call.

Our findings suggest that patients will encounter a range of psychosocial obstacles to work participation and there is a danger both they and their significant others will perceive these obstacles as insurmountable especially in the face of socio-cultural scepticism about their condition, along with widespread disparagement of the unemployed. Many disadvantaged individuals become entrenched in a position whereby it becomes all the more important to be seen as completely disabled. In adopting this stance and limiting their activity, the chances of any return to work and economic activity become increasingly remote. Importantly, our research suggests that well-intentioned support from those close to patients may be serving only to further entrench this position of total disability, adding to the body of previous evidence which demonstrates how solicitous responding by others to pain related behaviours can be associated with a range of negative outcomes [25]. Thus, the role of significant others may warrant further investigation by those looking to facilitate effective vocational rehabilitation. This is especially important in light of the on-going changes to the welfare system in the United Kingdom, which are being implemented to reduce its substantial cost burden, as it raises the possibility that initiatives will be interpreted as removal of support, being linked to and allied with punitive measures. Our findings suggest that it is possible that, where individuals are faced with more stringent tests and assessments to determine their eligibility for benefits, this may encourage further efforts by patients and those close to them to demonstrate the (perceived) severity and impact of the illness, thus entrenching attitudes and leading to further inactivity.

However, our findings also demonstrate that significant others are clearly important sources of support to individuals with back pain, and their inclusion in any such rehabilitation and education programmes could potentially be a valuable way of mobilising readily accessible resources in a way that supports optimal functioning. Indeed, it has been suggested that a ‘can do’ focus may be associated with better functioning in terms of work participation outcomes [35]. This approach is in line with current UK government policy and the introduction of the Statement of Fitness for Work (the fit note –http://www.dwp.gov.uk/fitnote) by the Department for Work and Pensions in April 2010. This replaced the previous Medical Statement (the sick note), and changed the focus firmly to what people can do despite their health problem, as opposed to emphasising (and certifying) what they cannot do.

It is acknowledged that a limitation of this study is the small sample of people with back pain, all recruited from one geographical area in the United Kingdom. Additionally, we recognise that the different social welfare arrangements in place in the different countries of the developed world may well impact on the extent to which these findings can be generalised to other international settings. Nevertheless, our use of in-depth qualitative research methods provides novel and potentially useful data on which further work can build. Many of our findings support and extend previous findings from work with long-term welfare claimants and further research focusing on individuals recruited from primary care settings may offer additional insight into the disability trajectory, identifying at what point interventions focused on significant others may be most valuable. Future research might also usefully explore in more depth the relative importance on the specific dimensions delineated in this study. This study cannot offer a perspective of those who report having no significant other, and we did not examine in any depth differences between significant other type. Whilst living on one’s own does not necessarily mean that one has no significant other support, it may be that there are differences worth examining between dyads based on whether or not they co-habit, and it might also be useful to explore different types of significant other (for example parent/ child dyads in comparison to spousal dyads).

## Conclusion

This study adds to a growing body of literature that highlights some of the complexities involved in determining work participation outcomes for individuals with back pain. In particular, this work draws attention to ways in which both close others and wider social circumstances may impact on functional outcomes, including work participation. Our findings suggest that the role of close others may be an area warranting careful consideration by those designing and implementing programmes intended to facilitate vocational rehabilitation. Findings from this study suggest that, to be effective, work participation programmes should be flexibly designed, able to assess and manage individuals and their wider social circumstances. Programmes intended to facilitate return to work also need to acknowledge the potential social stigma faced by both individuals with ‘unseen’ health complaints and their families, Such programmes should recognise that participants’ understandable attempts to achieve legitimisation of their symptoms may be acting as a barrier to full engagement and that any less adaptive illness beliefs on the part of individuals and their close others may need identifying and addressing.

## Abbreviations

CSM: Common-sense model of self-regulation.

## Competing interests

The authors declare that they have no competing interests.

## Authors’ contributions

All authors participated in the design and conception of the study. JB, SM and NK conducted the interviews. JB and SM read, coded and analysed the data. NK and KB checked the coding and analysis. JB drafted the manuscript and all authors read, revised and approved the final manuscript.

## Authors’ information

JB is a Research Fellow at the Centre for Applied Psychological Research, University of Huddersfield. Her primary research interests focus on the management of long term chronic illnesses and the influences of and impact on those close to patients.

SM is a Senior Research Fellow at the Centre for Health and Social Care Research, University of Huddersfield. Her primary research interests focus on the influence of the family on sickness absence and work disability.

NK is Professor of Applied Psychology and Director of the Centre for Applied Psychological Research at the University of Huddersfield. He is an expert in qualitative research methodologies and the creator of the particular style of qualitative analysis methodology (Template Analysis) used in this study.

KB is an occupational health consultant and clinical scientist, and visiting Professor at the Centre for Health and Social Care Research at the University of Huddersfield. He has been involved in the development of evidence-based clinical guidelines and public educational material for a range of musculoskeletal problems.

## Pre-publication history

The pre-publication history for this paper can be accessed here:

http://www.biomedcentral.com/1471-2474/14/48/prepub
